# Factors associated with severe maternal morbidity and near miss in the *São Francisco* Valley, Brazil: a retrospective, cohort study

**DOI:** 10.1186/1471-2393-14-91

**Published:** 2014-02-27

**Authors:** Alvaro José Correia Pacheco, Leila Katz, Alex Sandro Rolland Souza, Melania Maria Ramos de Amorim

**Affiliations:** 1 Federal University of the São Francisco Valley (UNIVASF), Avenida José de Sá Maniçoba, s/n, Centro, 56.304-917 Petrolina, PE, Brazil; 2 Postgraduate Program, Instituto de Medicina Integral Prof. Fernando Figueira (IMIP), Rua dos Coelhos, 300, Boa Vista, 50.070-902 Recife, PE, Brazil; 3 Obstetric Intensive Care Unit, Instituto de Medicina Integral Prof. Fernando Figueira (IMIP), Rua dos Coelhos, 300, Boa Vista, 50.070-902 Recife, PE, Brazil; 4 Federal University of Pernambuco (UFPE), Cidade Universitaria, Brazil; 5 Federal University of Campina Grande (UFCG), Campina Grande, Brazil

**Keywords:** Maternal mortality, Morbidity, Prenatal, Risk factors, Cohort studies

## Abstract

**Background:**

Maternal mortality remains a major public health issue worldwide, with persistent high rates prevailing principally in underdeveloped countries. The objective of this study was to determine the risk factors for severe maternal morbidity and near miss (SMM/NM) in pregnant and postpartum women at the maternity ward of the Dom Malan Hospital, Petrolina, in northeastern Brazil.

**Methods:**

A retrospective, cohort study was conducted to evaluate the sociodemographic and obstetric characteristics of the women. Patients who remained hospitalized at the end of the study period were excluded. Risk ratios (RR) and their respective 95% confidence intervals (95% CI) were calculated as a measure of relative risk. Hierarchical multiple logistic regression was also performed. Two-tailed p-values were used for all the tests and the significance level adopted was 5%.

**Results:**

A total of 2,291 pregnant or postpartum women receiving care between May and August, 2011 were included. The frequencies of severe maternal morbidity and near miss were 17.5% and 1.0%, respectively. Following multivariate analysis, the factors that remained significantly associated with an increased risk of SMM/NM were a Cesarean section in the current pregnancy (OR: 2.6; 95% CI: 2.0 – 3.3), clinical comorbidities (OR: 3.4; 95% CI: 2.5 – 4.4), having attended fewer than six prenatal visits (OR: 1.1; 95% CI: 1.01 – 1.69) and the presence of the third delay (i.e. delay in receiving care at the health facility) (OR: 13.3; 95% CI: 6.7 – 26.4).

**Conclusions:**

The risk of SMM/NM was greater in women who had been submitted to a Cesarean section in the current pregnancy, in the presence of clinical comorbidities, fewer prenatal visits and when the third delay was present. All these factors could be minimized by initiating a broad debate on healthcare policies, introducing preventive measures and improving the training of the professionals and services providing obstetric care.

## Background

Maternal mortality remains a major public health issue worldwide. Although reducing maternal mortality is part of the international commitment referred to as the Millennium Development Goals and despite the fact that the latest studies have suggested a trend towards a decrease in its incidence in recent years [1-3], rates remain high, with an unequal global distribution in which 99% of cases occur in underdeveloped countries [4-7].

Studies on the physiopathology of pregnancy, childbirth and the postpartum have revealed a wide spectrum of clinical conditions in women, ranging from a healthy pregnancy to the other extreme of maternal death. Severe maternal morbidity forms part of this range of clinical conditions and begins with the occurrence of a complication that could progress to maternal death. Another extremely critical group that merits particular emphasis concerns cases that are referred to as *near miss* which is a more severe condition than severe maternal morbidity [8,9].

Ever since the 1990s, the interest of investigators in patients who survive an experience of severe maternal morbidity or near miss has been increasing, since the study of such patients may permit extrapolations to be made on the quality of care provided to women during pregnancy and in the postpartum [10-29]. In the largest population-based study conducted to this date, which was carried out in the Netherlands and involved 371,021 women, the authors described a frequency of near miss of 0.7% of all deliveries performed in that country [10].

In 2009, the World Health Organization (WHO) developed an instrument to standardize the definition of cases of severe maternal morbidity and near miss with the aim of evaluating the quality of obstetric care. This instrument uses an association of three major groups of criteria: management complexity; signs, symptoms or specific clinical entities; and organ dysfunction [30].

Identifying both the clinical factors associated with states of maternal morbidity and the delays that occur in the sequence of the obstetric care provided may permit early intervention to be made within the process that leads a healthy pregnant or postpartum woman to the other extreme in this chain of events, i.e. to maternal death [31-40].

## Methods

A retrospective cohort study was conducted in the maternity ward of the Dom Malan Hospital, a reference center in obstetric care for high risk pregnancies in the São Francisco Valley region. Located in the town of Petrolina in the state of Pernambuco, it is the only reference center for high complexity obstetric and neonatal care within the region covered by the Integrated Development Program for the São Francisco Valley region of northeastern Brazil.

Sample size was calculated using the OpenEpi software program, version 2.3.1, after data had been collected from the first 600 women. These data indicated a rate of severe maternal morbidity/near miss of 20% for women who had been submitted to a Cesarean section in their previous pregnancy compared to 15% in women who had had a vaginal delivery. Considering a significance level of 95%, relative precision of 20% and an expected loss of 20%, sample size was established as 2,240 patients. This number was rounded up to 2,290, anticipating an initial programmed data collection period of four months.

A total of 2,291 pregnant or postpartum women, consecutively admitted to the Dom Malan Hospital between May and August 2011, were included in the study. Those who had not yet been discharged within the study period or whose medical records were unavailable were excluded from the study. Since the data were collected after the patients had been discharged from hospital, the institute’s internal review board was asked to waive the requirement for informed consent.

The study was initiated only after approval of the protocol had been granted by the internal review board of the Federal University of the *São Francisco* Valley (UNIVASF) in approval letter # 0039.0.441.000-11.

Severe maternal morbidity (SMM) and near miss (NM) are defined as the presence of at least one of the criteria adopted by the World Health Organization (WHO) in 2009 [30]. For the purpose of this study, we chose to evaluate severe maternal morbidity and near miss maternal mortality as a group. The term SMM/NM is used to describe patients who have suffered severe maternal morbidity and/or a near miss. The sociodemographic and obstetric characteristics of the women were evaluated, as well as the presence of any of the “three delays”. Co-morbidities were considered present when there were preexisting medical conditions or chronic conditions recognized during pregnancy that could negatively affect its course for example hypertension, diabetes, maternal HIV infection or heart disease.

The “three delays” model was originally conceived to evaluate cases of maternal death, [11] and was later adopted for the study of cases of near miss. The first delay consists of a situation in which the patient or her family fails to recognize the severity of her situation and delays the decision to seek care. The second delay refers to the patient’s inability to gain access to a healthcare service after having made the decision to seek care. The third delay concerns the inability of the healthcare professionals to provide patients with adequate or timely care, or a lack of basic infrastructure within the healthcare service to meet the demands made on it [11].

Prior to initiating data collection, three researchers involved were trained to identify the delays through the analysis of information from medical charts. Three observers assessed the files and evaluated the presence of delays, and if there were different opinions the decision of two of the reviewers prevailed. Regarding the prenatal care first delay was considered present if prenatal care was absent, if the number of prenatal care visits was below the recommendations of Brazil Ministry of Health, or if prenatal care was started after the first trimester. A first delay was considered present also, if medical chart informed that despite the presence of conditions that indicate the need of medical attention, the patient did not seek for care. A second delay was considered if there was information that the patient or her family tried to reach medical assistance but did not have access. Third delays were identified according to data recorded on medical charts regarding the management given to each case. This management was compared to protocols provided by the Brazilian Federation of Societies of Obstetrics and Gynecology (FEBRASGO), by FIGO (International Federation of Gynecology and Obstetrics) and the World Health Organization (WHO) and judged that a third delay was present if there was an inadequate management or a delay in adequate conduction.

The statistical analysis was performed using the Epi-Info software program, version 7 (Atlanta, GA, USA). Frequencies were calculated for the categorical variables, and means and their respective standard deviations were calculated for the numerical variables. A bivariate analysis was conducted to verify the existence of differences between the groups using Pearson’s chi-square test or Fisher’s exact test, as appropriate. All p-values were two-tailed and a significance level of 5% was adopted. To determine the strength of the association, risk ratios (RR) and their 95% confidence intervals (95% CI) were calculated.

All the variables evaluated in the bivariate analysis were included in the multiple logistic regression analysis to identify those most strongly associated with SMM/NM, thus determining the adjusted risk. The causal model was adopted and the variables were divided into blocks, the more distal factors being the biological and socioeconomic variables (level 1), followed by the reproductive, prenatal and lifestyle variables (level 2) and, finally, the variables concerning the presence of comorbidities, whether the patient had been submitted to a Cesarean section and the presence of the three delays (level 3), these being considered the variables closest to the end-point. Stepwise logistic regression was performed, with the variables associated with the end-point at a significance level of 20% remaining in the model. The procedure was then repeated with these variables for a significance level of 5%, and a final regression was then performed to determine the adjusted risk of SMM/NM for each one of the variables significantly associated with the end-point at a significance level of 5%.

## Results

A total of 2,291 patient charts were analyzed, with three maternal deaths being identified. In the other 2,288 charts, 400 cases of severe maternal morbidity (17.5%) were found, as well as 24 records containing criteria indicative of a near miss (1%).

The mean age of the patients with SMM/NM was 25.4 ± 7.1 years compared to 24.2 ± 6.5 years for the other patients (p = 0.001). The patients with SMM/NM had a mean of 8.2 years of schooling and a median of two pregnancies, while median parity was one. Mean gestational age at admission was 35.4 ± 6.1 weeks, with deliveries occurring at a mean of 36.5 ± 5.7 weeks. The median number of prenatal visits attended by the patients with SMM/NM was 6.0. Mean birth weight of the newborn infants of the women with SMM/NM was lower (2,811 ± 855.8 grams) compared to the infants of the women without these complications (3,130 ± 586.6 grams) (p < 0.0001) (Table 1).

**Table 1:** Characteristics of the patients with severe maternal morbidity (SMM) and near miss (NM)

**Characteristic**	**SMM/NM**				**p-value** *
	**Present**		**Absent**		
	**(n = 400)**		**(n = 1888)**		
Age in years (mean/SD)	25.4	7.1	24.2	6.5	0.001
Schooling in years (mean/SD)	8.2	3.4	8.0	3.1	0.24
Number of pregnancies (Median/IQR)	2.0	1-3	2.0	1-3	0.18
Number of deliveries (Median/IQR)	1.0	0-2	1.0	0-2	0.12
Gestational age at admission in weeks (mean/SD)	35.4	6.1	35.7	8.3	0.43
Gestational age at delivery in weeks (mean/SD)	36.5	5.7	36.1	8.1	0.38
Number of prenatal visits (Median/IQR)	6.0	4-8	6.0	5-8	0.22
Birth weight in grams (mean/SD)	2.811	855.8	3.130	586.6	<0.0001

*Mann Whitney test; SD: standard deviation; IQR: interquartile range.

In the bivariate analysis, statistically significant associations were found between the following characteristics and SMM/NM: history of a previous Cesarean section (RR: 1.43; 95% CI: 1.13 – 1.81; p = 0.003), cesarean section in the current pregnancy (RR: 1.99; 95% CI: 1.71-2.28; p < 0.0001), presence of the third delay (RR: 4.0; 95% CI: 3.27 – 4.97; p < 0.0001), presence of the second delay (RR: 3.4; 95% CI: 2.41 – 4.70; p < 0.0001), presence of clinical comorbidities in general (RR: 2.7; 95% CI: 2.31 – 3.26; p < 0.0001) and of chronic arterial hypertension in particular (RR: 6.78; 95% CI: 2.26 – 20.33; p < 0.0001) (Table 2).

**Table 2:** Factors associated with severe maternal morbidity (SMM) and near miss (NM) in the bivariate analysis

**Variable**	**SMM/NM**		**RR**	**95% CI**	**p-value**
	**Present (n = 400)**				
	**n**	**%**			
< 4 years of schooling					
**Yes**	32	20.5	1.21	0.88-1.69	0.23*
**No**	303	16.8			
Age < 15 years					
**Yes**	8	14.5	0.83	0.43-1.58	0.56*
**No**	391	17.5			
Age > 35 years					
**Yes**	34	21.5	1.27	0.93-1.74	0.14*
**No**	365	17.1			
Skin color black					
**Yes**	15	22.7	1.31	0.83-2.06	0.25*
**No**	385	17.3			
No partner					
**Yes**	184	17.9	1.05	0.88- 1.25	0.60*
**No**	216	17.1			
<6 prenatal visits					
**Yes**	136	18.2	1.13	0.93-1.38	0.20*
**No**	202	16.0			
Number of pregnancies ≤ 4					
**Yes**	83	18.4	1.09	0.88-1.37	0.40*
**No**	302	16.8			
Covered by the national health system					
**Yes**	274	17.0	0.90	0.74-1.09	0.31*
**No**	126	18.7			
Prior Cesarean section					
**Yes**	67	22.9	1.43	1.13-1.81	0.003*
**No**	290	16.0			
Cesarean section in current pregnancy					
**Yes**	181	29.7	1.99	1.71-2.28	<0.0001*
**No**	219	13.1			
Presence of any comorbidity					
**Yes**	145	36.6	2.74	2.31-3.26	<0.0001*
**No**	255	13.5			
Chronic arterial hypertension #					
**Yes**	73	52.1	6.78	2.26-20.33	<0.0001*
**No**	3	7.7			
Diabetes ##					
**Yes**	6	26.1	2.73	0.86-8.72	0.08**
**No**	4	9.5			
Maternal obesity ###					
**Yes**	1	50.0	1.78	0.13-10.13	0.08 **
**No**	4	9.5			
Smoking					
**Yes**	10	12.8	1.25	0.42-3.73	0.68**
**No**	4	10.3			
First delay					
**Yes**	119	18.4	1.07	0.88-1.30	0.48*
**No**	281	17.1			
Second delay					
**Yes**	16	57.1	3.36	2.41-4.70	<0.0001*
**No**	380	17.0			
Third delay					
**Yes**	38	53.5	4.04	3.27-4.97	<0.0001*
**No**	362	16.2			

*Chi-square test **Fisher’s exact test. #Data available for only 179 women. ##Data available for only 65 women. ###Data available for only 44 women.

After multivariate analysis, the following characteristics remained significantly associated with severe maternal morbidity and/or near miss: a Cesarean section in the current pregnancy (OR: 2.6; 95% CI: 2.00 – 3.35), the presence of any comorbidity (OR: 3.4; 95% CI: 2.57 – 4.40), presence of the third delay (OR: 13.3; 95% CI: 6.73 – 26.37) and having attended fewer than six prenatal visits (OR: 1.13; 95% CI: 1.01 – 1.69) (Table 3).

**Table 3:** Conditions associated with severe maternal morbidity (SMM) and near miss (NM) following multivariate analysis

**Variable**	**Coefficient**	**Standard error**	**Odds ratio**	**95% CI**	**p-value**
**Cesarean section in current pregnancy**	0.95	0.13	2.6	2.00 – 3.35	<0.0001
**Presence of comorbidities**	1.20	0.13	3.4	2.57- 4.40	<0.0001
**< 6 prenatal visits**	0.27	0.13	1.13	1.01-1.69	0.04
**Third delay**	2.59	0.34	13.3	6.73-26.37	<0.0001

## Discussion

In the present study, the frequency of severe maternal morbidity and near miss was high compared to that found in developed countries [2,10], considering that 17.5% of the women were found to have at least one of the conditions defining SMM and 1% had criteria indicative of a near miss during the period evaluated [10,15,23-28]. A recent systematic literature review published in 2012 on this subject indicated a prevalence that ranged from 0.04% to 15% depending on the criteria used to define it [1].

During the study period, three maternal deaths occurred in the Dom Malan Hospital, revealing a relatively low near miss/maternal mortality ratio (1.8), in agreement with the findings of a nationwide study published in 2012 [32], but in conflict with the results found in studies conducted in developed countries [1,15,18,36], including a study carried out in the Netherlands that reported a ratio of one death for every 53 cases of near miss [10].

In addition to the variation in the actual incidence of SMM and NM in the different regions, the numbers may also be affected by the different definitions used for these conditions, which may explain the substantial variations in the frequency of SMM/NM found in the literature. The present study used the criteria proposed by the World Health Organization in 2009 [30], whereas other studies have used different defining criteria such as those established by Mantel or Waterstone [22,26,27].

Some studies have reported the extreme limits of reproductive age as being a condition associated with a greater risk of SMM/NM [12,26,29]; however, this was not found in the present study. It is possible that the regional characteristics of the population may have influenced the results. There were relatively few women at the extreme limits of reproductive age in this study, with women under 15 years of age accounting for only 3.5% of the sample, while those over 35 years of age accounted for 7% of the sample. This may have limited the power of the study to evaluate these groups of women.

Gestational age at the time of delivery being within the definitions of prematurity, 1^st^ minute Apgar score < 7 and birth weight < 2,500 grams in patients with SMM/NM may reflect a need to intervene in the natural progression of diseases associated with unfavorable outcomes, particularly in the case of hypertensive syndromes [20,21,25,28,32]. The fact that the Dom Malan Hospital is the only service in the region with neonatal and obstetric intensive care units (ICU) explains the large number of premature infants and pregnant women with comorbidities or obstetric complications in whom early delivery is a common form of management. In the present study, 12.7% of the patients suffered hypertensive complications, which may in part explain these findings.

Evaluating the factors associated with the occurrence of SMM/NM, multiple logistic regression analysis showed that only the variables *having been submitted to a Cesarean section in the current pregnancy*, *presence of comorbidity*, *having attended fewer than six prenatal visits* and *the presence of the third delay* significantly increased this risk.

In relation to having been submitted to a Cesarean section in the current pregnancy, the extreme importance of this issue merits particular emphasis in view of the high and continually rising rates of this intervention in women in Brazil, reaching 52.0% of deliveries according to data published by the Ministry of Health in 2010 [41]. In the present study, having been submitted to a Cesarean section in the current pregnancy was associated with a 2.5-fold greater risk of experiencing severe maternal morbidity or a near miss. This finding is in agreement with data published from other studies [25,38]. In a study involving approximately 370,000 Dutch women, a relative risk of 5.2 was found for progression to near miss in patients who had been submitted to a Cesarean section in a previous pregnancy and of 5.9 for women submitted to an elective Cesarean section in the current pregnancy [10]. It appears reasonable to speculate that the intrinsic morbidity associated with being submitted to this procedure, including the higher risk of infection, hemorrhage, thromboembolism or complications, justifies the results found.

Being aware of the sequence of events that may occur during the course of a normal pregnancy leading to the other extreme of maternal death contributes towards understanding the association found between the presence of comorbidities and SMM/NM [8]. The results of the present study show a significant increase in the risk of progression to SMM/NM in women with a history of arterial hypertension prior to becoming pregnant. This finding is in agreement with reports from various similar studies in which hypertensive syndromes during pregnancy were more common not only in patients with SMM/NM but also in those cases that resulted in maternal death [2,20,21,25,28,32]. In a study conducted in a tertiary maternity hospital in the city of Campinas in the Brazilian state of São Paulo, a rate of hypertension of 96% was found in patients with severe maternal morbidity [23]. Other studies have concluded that hypertensive syndromes remain a significant cause of mortality in pregnant women, constituting the principal cause of direct obstetric maternal mortality in various locations [19,20].

The median number of prenatal visits attended by the patients with SMM/NM in the present study was in accordance with the recommendations of the Brazilian Ministry of Health, i.e. at least six visits [42]. This finding reflects the increase in prenatal coverage at primary healthcare level in Brazil, probably as a result of the expansion in the teams working in the family health strategy [38]. On the other hand, 14% of the patients seen at the Dom Malan Hospital at the time of this study had attended fewer than six prenatal visits, a condition that is significantly associated with an increased risk of morbidity, as shown in a study conducted in the city of Niteroi, Rio de Janeiro, in 2009 in which 30% of the women identified with severe maternal morbidity according to the WHO criteria had not attended any prenatal visits [28].

Another fact that merits attention is that only 5.7% of the patients admitted to the Dom Malan Hospital were referred from primary healthcare, with the majority of the patients seeking the service spontaneously (69.2%). Although the influence of the different healthcare levels (primary, secondary and tertiary) was not analyzed in the present study, these results show a greater frequency of patient-related delay (the first of the delays in the “three delays model”) in the group of women with SMM/NM [11]. However, following multivariate analysis, this variable did not remain significantly associated with the end-point.

On the other hand, the delay associated with healthcare professionals, either in recognizing the situation of risk or in intervening in a timely manner (the third delay), remained significantly associated with a greater risk of developing SMM/NM, a finding that is in agreement with other recent studies [10,12,39,43]. The association between the third delay and SMM/NM is understandable, since the timely recognition of conditions that could develop unfavorably during pregnancy, childbirth or in the postpartum, and the provision of the necessary care in an opportune manner constitute the latest tool for interrupting the process that leads to SMM/NM. When all the previous measures fail and the woman is found to be in a situation of risk, the third delay may then contribute significantly to her progression to severe maternal morbidity.

Based on our review of the Pubmed, Embase, Lilacs/SciELO, Scopus and Cochrane databases, to the best of our knowledge this study is the first to be conducted on the subject of SMM/NM in the São Francisco Valley region of northeastern Brazil. This is important, bearing in mind that the indicators of maternal mortality are unsatisfactory in northeastern Brazil, specifically in the region in which this study was conducted, [41,42] while identifying the factors associated with SMM/NM may contribute towards implementing important changes in this scenario.

Furthermore, few international studies have evaluated the factors associated with the presence of severe maternal morbidity or near miss. At national level, this study is one of the first to evaluate this matter, thus adding significantly to the current knowledge available on the subject. The results found may help establish public healthcare policies and strategies aimed at tackling the issue of maternal morbidity and mortality.

The fact that this is a retrospective study in which the data were collected from patient records constitutes a limitation, since the charts may have been filled out inaccurately. Some of the variables collected initially and planned to be included in the causal theoretical model were excluded from the analysis because the data were unavailable, including such variables as, for example, the patient’s weight, how labor began, the association between the outcome and assisted reproduction, or the patient’s family income. Other variables which are risk factors for cesarean section and SMM/NM such as obstructed labour, placenta praevia, chorioamnionitis and placental abruption, were not available for analysis which also imposes a limitation to the study. Nevertheless, a large number of patients were included, guaranteeing the power of the sample to show relevant differences for the different risk factors investigated.

A future prospective study should be conducted to acquire further information on the profile of these patients and on the risk factors for SMM/NM, including a qualitative evaluation of the situation of patients affected by these events. Findings from such a study would contribute towards clarifying some of the gaps that remain in current knowledge. Listening to the women who have experienced events severe enough to be classified as SMM or NM will certainly form an important part of future studies.

## Conclusions

These results show that severe maternal morbidity and near miss occurred in a significant number of patients at the Dom Malan Hospital between May and August 2011. The principal risk factors associated with these conditions were the presence of clinical comorbidities, having had a Cesarean section, having attended fewer than six prenatal visits and the presence of delays associated with the healthcare professionals.

## Abbreviations

SMM: Severe maternal morbidity; NN: Near miss; WHO: World Health Organization; RR: Relative risk; CI: Confidence interval; UNIVASF: *Universidade Federal do Vale do São Francisco/*Federal University of the *São Francisco* Valley; ICU: Intensive care unit; IQR: Interquartile range; SD: Standard deviation.

## Competing interests

The authors declare that they have no competing interests.

## Authors’ contributions

AJCP was responsible for having designed the study together with LK, MMRA and ARSS. AJCP collected the data. AJCP, LK and ARSS conducted the analysis and wrote the first draft of the manuscript under the supervision of MMRA. LK and MMRA reviewed the article and all the authors read and approved the final manuscript.

## Pre-publication history

The pre-publication history for this paper can be accessed here:

http://www.biomedcentral.com/1471-2393/14/91/prepub
